# The Anti-Inflammatory and Pain-Relieving Effects of AR170, an Adenosine A_3_ Receptor Agonist, in a Rat Model of Colitis

**DOI:** 10.3390/cells9061509

**Published:** 2020-06-21

**Authors:** Luca Antonioli, Elena Lucarini, Catia Lambertucci, Matteo Fornai, Carolina Pellegrini, Laura Benvenuti, Lorenzo Di Cesare Mannelli, Andrea Spinaci, Gabriella Marucci, Corrado Blandizzi, Carla Ghelardini, Rosaria Volpini, Diego Dal Ben

**Affiliations:** 1 Department of Clinical and Experimental Medicine, University of Pisa, 56126 Pisa, Italy; lucaant@gmail.com (L.A.); mfornai74@gmail.com (M.F.); laura.benvenuti962@gmail.com (L.B.); c.blandizzi@gmail.com (C.B.); 2 Department of Neurosciences, Psychology, Drug Research and Child Health–Neurofarba–Section of Pharmacology and Toxicology, University of Florence, 50139 Florence, Italy; elena.lucarini@unifi.it (E.L.); lorenzo.mannelli@unifi.it (L.D.C.M.); carla.ghelardini@unifi.it (C.G.); 3 School of Pharmacy, Medicinal Chemistry Unit, University of Camerino, 62032 Camerino (MC), Italy; catia.lambertucci@unicam.it (C.L.); andrea.spinaci@unicam.it (A.S.); gabriella.marucci@unicam.it (G.M.); diego.dalben@unicam.it (D.D.B.); 4 Department of Pharmacy, University of Pisa, 56126 Pisa, Italy; carolina.pellegrini@gmail.com

**Keywords:** A_3_ adenosine receptors, immune cells, experimental colitis, DNBS, visceral pain, oxidative stress

## Abstract

The pharmacological activation of A_3_ receptors has shown potential usefulness in the management of bowel inflammation. However, the role of these receptors in the control of visceral hypersensitivity in the presence of intestinal inflammation has not been investigated. The effects of AR170, a potent and selective A_3_ receptor agonist, and dexamethasone (DEX) were tested in rats with 2,4-dinitrobenzene sulfonic acid (DNBS)-induced colitis to assess their tissue inflammatory parameters. The animals received AR170, DEX, or a vehicle intraperitoneally for 6 days, starting 1 day before the induction of colitis. Visceral pain was assessed by recording the abdominal responses to colorectal distension in animals with colitis. Colitis was associated with a decrease in body weight and an increase in spleen weight. The macroscopic damage score and tissue tumor necrosis factor (TNF), interleukin 1β (IL-1β), and myeloperoxidase (MPO) levels were also enhanced. AR170, but not DEX, improved body weight. Both drugs counteracted the increase in spleen weight, ameliorated macroscopic colonic damage, and decreased TNF, IL-1β, and MPO tissue levels. The enhanced visceromotor response (VMR) in rats with colitis was decreased via AR170 administration. In rats with colitis, AR170 counteracted colonic inflammatory cell infiltration and decreased pro-inflammatory cytokine levels, thereby relieving visceral hypersensitivity.

## 1. Introduction

Inflammatory bowel diseases (IBDs), including Crohn’s disease and ulcerative colitis, are complex multifactorial inflammatory diseases of the gut, driven by genetic, luminal, and environmental factors, leading to an overactive intestinal immune response [1]. Typically, IBDs are characterized by diarrhea or constipation, nausea, weight loss, and rectal bleeding [1].

The visceral pain experienced by 50–70% of IBD patients is one of the most significant problems for subjects suffering from these chronic illnesses [2]. Pain and abdominal discomfort represent a significant burden in these patients that diminishes their quality of life [3]. Indeed, although majority of patients suffering from acute flares of IBD experience pain, which is typically improved by a decrease in disease activity, a significant percentage of IBD patients continue to perceive pain despite resolving their inflammation and achieving clinical remission [2]. Based on these premises, the identification of new therapeutic targets for developing novel pharmacological tools able to manage both the immuno–inflammatory components of IBD and to curb visceral sensitivity represents a significant medical need.

A large body of evidence has highlighted the involvement of adenosine in the maintenance of intestinal homeostasis, which orchestrates the interplay between the intestinal epithelial cells, the neuromuscular compartment, and the enteric immune system [4,5,6,7,8]. In particular, adenosine, via the engagement of four G protein-coupled receptors (named A_1_, A_2A_, A_2B_, and A_3_ receptors (A_1_AR, A_2A_AR, A_2B_AR, and A_3_AR, respectively)), plays a key role in driving an immune response [5]. Among them, A_3_AR has strongly captured the interest of the scientific community, since increasing evidence reveals the complex role of this receptor subtype in the pathophysiology of inflammation, as A_3_AR participates in the modulation of a broad array of immune cell functions, such as cytokine production, degranulation, chemotaxis, and proliferation [7,9,10].

**Figure 1:**
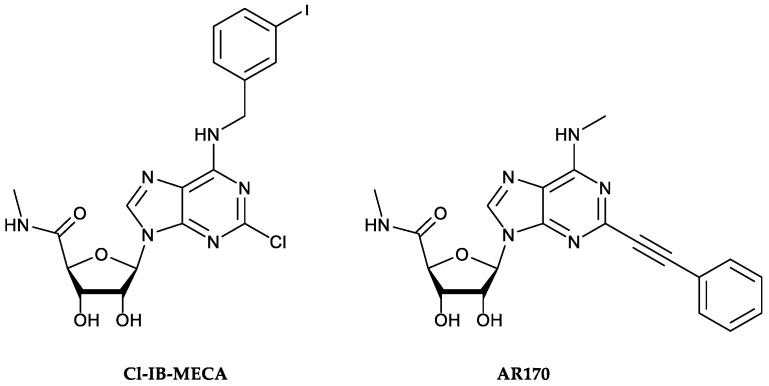
Chemical structures of the A_3_AR reference agonist 2-chloro-*N*^6^-(3-iodobenzyl)-adenosine-5′-*N*-methyluronamide (Cl-IB-MECA, **left**) and the potent and selective A_3_AR agonist AR170 (**right**) employed in this study.

Over the years, these receptors have also revealed an involvement in the pathophysiology of IBDs [11]. Indeed, preclinical and clinical studies demonstrated a marked alteration of A_3_AR expression in these conditions, accompanied by increased production of pro-inflammatory cytokines [11,12]. The pharmacological engagement of A_3_AR determines the inhibition of several cytokine/chemokine/inflammatory genes, thus promoting a marked down-regulation of several pro-inflammatory mediators (i.e., IL-1, IL-6, IL-12, Macrophage Inflammatory Protein 1α (MIP-1α), and MIP-2), as well as the production of oxidative stress, thereby improving experimental colitis. Furthermore, recent work by Ren et al. [12] demonstrated that 2-chloro-*N*^6^-(3-iodobenzyl)-adenosine-5′-*N*-methyluronamide (Cl-IB-MECA, Figure 1), a selective A_3_AR agonist, can inhibit the NF-κB pathway in the colonic epithelia of dextran sulfate sodium (DSS) colitis mice [12].

Several authors have highlighted the potential anti-nociceptive effects of A_3_AR activation [13,14]. For instance, Hou et al. [13] described the involvement of A_3_ARs in the beneficial effects exerted by electroacupuncture on the hypersensitivity induced by colitis in mice [13]. Similarly, Coppi et al. [14] demonstrated an A_3_AR activation-mediated pain-relieving mechanism involving the *N*-type Ca^2+^ channel block and action potential inhibition in the dorsal root ganglion neurons isolated from controls, as well as from animals treated with DNBS. This effect is consistent with the acute visceral pain relief showed by A_3_AR agonists in rats [15]. However, the potential therapeutic effect of the A_3_AR agonist on visceral pain development and persistence is an aspect that needs to be further investigated. In parallel, the stimulation of A_3_AR ameliorated the colonic motor disturbances associated with intestinal inflammation [16], thereby corroborating the relevance of this receptor subtype as an intriguing target for the management of IBDs.

Based on these premises, our study was designed to evaluate the effect of AR170 (Figure 1) [17,18,19], a potent and selective A_3_AR agonist, in counteracting the inflammatory process and curbing visceral hypersensitivity in a murine model of DNBS-induced intestinal inflammation.

## 2. Materials and Methods

### 2.1. Animals

Albino male Sprague–Dawley rats, 250–300 g in body weight, were employed throughout the study. The animals were fed standard laboratory chow and tap water ad libitum and were not subjected to experimental procedures for at least one week after their delivery to the laboratory. Animal care and handling were performed in accordance with the provisions of the European Community Council Directive 2010/63/UE, which were recognized and adopted by the Italian Government. All experimental procedures were approved by the Ethical Committee for Animal Experimentation of the University of Pisa and by the Italian Ministry of Health (authorization n° 674/2016-PR). In the present study, animal data are presented according to ARRIVE guidelines.

### 2.2. Induction of Colitis and Drug Treatments

Colitis was induced in accordance with the method previously described by Antonioli et al. [20]. Briefly, during a short period of anesthesia with isoflurane (Abbott, Rome, Italy), 30 mg of DNBS in 0.25 mL of 50% ethanol was administered intrarectally via a polyethylene PE-60 catheter inserted 8 cm proximal to the anus. Control rats received 0.25 mL of 50% ethanol. Animals underwent subsequent experimental procedures 6 days after DNBS administration to allow the full development of evident colonic inflammation. Test drugs were administered intraperitoneally for 6 days, starting 1 day before the induction of colitis. Animals were assigned to the following treatment groups: AR170 (3 mg/kg/day) or DEX (1 mg/kg/day). A group of animals was administered contextually with the A_3_AR antagonist MRS1523 (8 mg/kg i.p.) and AR170.

The acute effect of AR170 on visceral pain was assessed 14 days after DNBS injection, when pain persisted despite remission from colitis [21]. AR170 (0.5–4.5 mg/kg i.p.) was intraperitoneally administered 15 min before starting the test. The A_3_AR antagonist MRS1523 (8 mg/kg i.p.) was injected 15 min before AR170. To evaluate its effects on the development and persistence of the visceral hyperalgesia induced by DNBS, AR170 (1.5 mg/kg/day) was intraperitoneally administered for 14 days, starting from the day of DNBS injection, and tests were performed on days 8 and 15, 24 h after the last treatment.

DNBS-untreated animals (control group) and DNBS-treated rats (DNBS group) received only the drug vehicle. Body weight was monitored daily starting from the onset of drug treatments. All the evaluated parameters were not significantly affected in the DNBS-untreated animals administered with AR170 alone in comparison to the control group.

### 2.3. Assessment of Colitis

At the end of treatments, colonic tissues were excised, rinsed with saline, and scored for macroscopic and histological damage, in accordance with the criteria previously reported by Antonioli et al. [22]. The macroscopic criteria were scored on a 0–6 scale using the scoring system reported in Table 1. The presence of adhesions between colonic tissue and other organs (0 none, 1 minor, and 2 major adhesions) and the consistency of colonic fecal material (0 formed, 1 loose, and 2 liquid stools) were also scored [22]. All parameters of macroscopic damage were recorded and scored for each rat by two observers blinded to the treatment. At the time of experiment, the weight of the spleen was also measured.

### 2.4. Determination of Tissue Myeloperoxidase

MPO levels in colonic tissues were determined as previously reported by Antonioli et al. [20] and applied as a quantitative index to estimate the degree of mucosal infiltration by polymorphonuclear cells [20]. Briefly, the colonic tissue samples (300 mg) were homogenized 3 times (30 s each) at 4 °C with a polytron homogenizer (Cole Parmer Homogenizer, Vernon Hills, IL, USA) in 1 mL of ice-cold 50 mmol/L phosphate buffer (pH 6.0) containing 0.5% of hexadecyltrimethylammonium bromide to prevent the pseudoperoxidase activity of hemoglobin, as well as to solubilize membrane-bound MPO. The homogenate was sonicated for 10 s, frozen–thawed 3 times, and spun by centrifugation for 20 min at 18,000× *g*. The supernatant was then recovered and used for determination of MPO by means of a kit for an enzyme-linked immunosorbent assay (Bioxytech, Oxis International Inc., Portland, OR, USA). All samples were assayed within 2 days from collection. The results were expressed as the ng of MPO per 100 mg of tissue.

### 2.5. Cytokine Assays

Tissue TNF and IL-1β levels were measured with enzyme-linked immunosorbent assay kits (BioSource International, Camarillo, CA, USA) [23,24]. For this purpose, tissue samples, stored previously at 80 °C, were weighed, thawed, and homogenized in 0.3 mL of phosphate-buffered saline (PBS), pH 7.2/100 mg of tissue, at 4 °C and centrifuged at 13,400× *g* for 20 min. Aliquots (100 µL) of the supernatants were then used for the assay. Tissue TNF and IL-1β levels were expressed as the picogram per milligram of tissue or nanogram per milligram of tissue, respectively.

### 2.6. Assessment of Visceral Sensitivity

The extent of the abdominal contractions (VMR) due to colorectal distension was measured by performing electromyography (EMG) on the abdominal muscles and used as a quantitative measure of visceral sensitivity in the rats. Two EMG electrodes were sutured into the external oblique abdominal muscles of the animals under anesthesia and exteriorized dorsally [25]. VMR assessment was carried out under light anesthesia (2% isoflurane). A lubricated latex balloon (length: 4.5 cm) was attached on an embolectomy catheter and connected to a water-filled syringe used to perform colorectal distension (CRD). The syringe was used to fill the balloon placed into the colon with increasing volumes of water (0.5, 1, 2, and 3 mL, referred to as the distension volume). After colorectal stimulation, the EMG signal was recorded, amplified and filtered (Animal Bio Amp, ADInstruments, Colorado Springs, CO, USA), digitized (PowerLab 4/35, ADInstruments), analyzed, and quantified using LabChart 8 (ADInstruments). To quantify the VMR magnitude under each distension volume, the area under the curve (AUC) immediately before distension (30 s) was subtracted from the AUC during balloon distension (30 s), and the responses were expressed as a percentage increase from the baseline. The time elapsed between two consecutive distensions was 5 min. The entire measurement process lasted about 25 min.

### 2.7. Drugs and Reagents

Dimethyl sulfoxide, DNBS, DEX, MRS1523, and methylcellulose were purchased from Sigma-Aldrich (St. Louis, MO, USA). The synthesis of AR170 was performed as previously reported [17].

### 2.8. Statistical Analysis

The results are presented as the mean ± S.E.M. unless otherwise stated. The significance of differences was evaluated in the raw data by a one-way analysis of variance followed by a post hoc analysis via a Student–Newman–Keuls test or Bonferroni’s test. *p*-values < 0.05 were considered significantly different. All statistical procedures were performed using commercial software (GraphPad Prism, version 7.0 from GraphPad Software Inc., San Diego, CA, USA). Visceral sensitivity data were analyzed using the “Origin 9” software (OriginLab, Northampton, MA, USA).

## 3. Results

### 3.1. Body Weight and Spleen Weight

**Figure 2:**
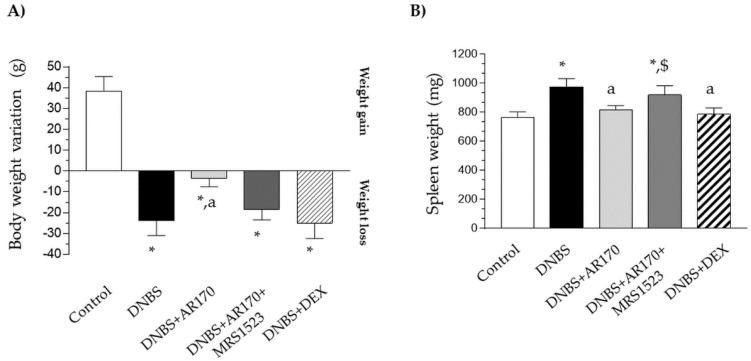
The effect of AR170 (3 mg/kg/day), alone or in combination with MRS1523 (8 mg/kg/day) or dexamethasone (DEX; 1 mg/kg/day), on body weight (**A**) and spleen weight; (**B**) at day 6 after the induction of colitis with 2,4-dinitrobenzene sulfonic acid (DNBS). Each column represents the mean ±SEM (*n* = 8–10). * *p* < 0.05, significant difference vs. the control group; ^a^ *p* < 0.05, significant difference vs. the DNBS group; ^$^ *p* <0.05, significant difference vs. the DNBS+AR170 group.

Six days after DNBS administration, the rats displayed a significant decrease in their body weight in comparison with the control animals (Figure 2A). Treatment with AR170 significantly counteracted the body weight decrease observed in the rats with colitis, whereas the animals subjected to dexamethasone administration did not experience this phenomenon (Figure 2A). The induction of colitis was also characterized by a significant increase in spleen weight. This increase was counteracted by AR170 and via dexamethasone administration (Figure 2B).

### 3.2. Colonic Length and Macroscopic Damage Score

**Figure 3:**
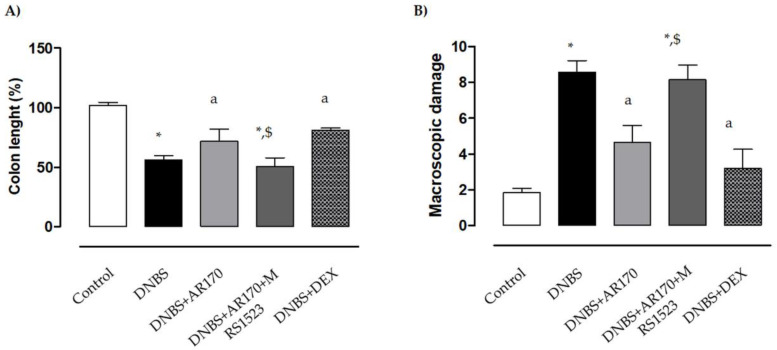
Effects of AR170 (3 mg/kg/day), alone or in combination with MRS1523 (8 mg/kg/day) or DEX (1 mg/kg/day), on colon length (**A**) and the macroscopic damage score (**B**) at day 6 after the induction of colitis with DNBS. Each column represents the mean ±S.E.M. (*n* = 8–10). * *p* < 0.05, significant difference vs. control group; ^a^ *p* < 0.05, significant difference vs. DNBS group; ^$^ *p* < 0.05, significant difference vs. DNBS + AR170 group.

Six days after DNBS administration, the inflamed rats were characterized by a shortening of colonic length (−43.7%) compared to the control animals. Treatment of the inflamed rats with the A_3_AR agonist AR170 or DEX significantly attenuated the decrease in colonic length (Figure 3A).

The administration of DNBS was associated with colonic thickening and ulcerations, with marked areas of transmural inflammation. Moreover, adhesions and bowel dilations were detected, with macroscopic damage accounting for 8.6 ± 0.8. In this setting, the macroscopic damage was reduced significantly by AR170 and DEX (Figure 3B). MRS1523 counteracted the effects of AR170 (Figure 3A,B), but not dexamethasone efficacy (not shown).

### 3.3. MPO Levels in Colonic Tissues

**Figure 4:**
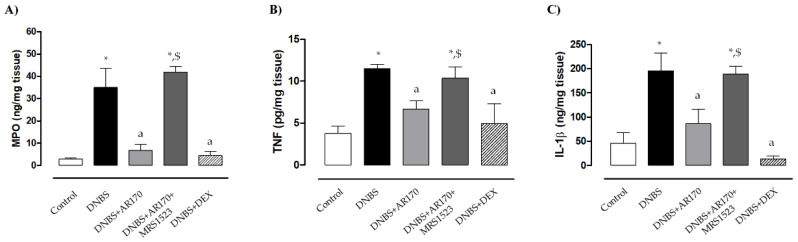
Myeloperoxidase (MPO) (**A**), tumor necrosis factor (TNF) (**B**), and interleukin 1β (IL-1β) levels (**C**) in colonic tissue in the control rats and in animals treated with DNBS alone or in combination with AR170 (3 mg/kg/day), AR170 plus MRS1523 (8 mg/kg/day), or DEX (1 mg/kg/day). Each column represents the mean ±S.E.M. (*n* = 8–10). * *p* < 0.05, significant difference vs. control rats; ^a^ *p* < 0.05, significant difference vs. DNBS group; ^$^ *p* < 0.05, significant difference vs. the DNBS + AR170 group.

Rats with DNBS-induced colitis showed a marked increase in colonic MPO levels (35 ± 8.6 ng/mg tissue) compared to the control animals (2.8 ± 0.5 ng/mg tissue). Treatment with all test drugs significantly prevented the increase in colonic MPO levels associated with DNBS administration (Figure 4A). MRS1523 significantly counteracted AR170 (Figure 4A) but not the dexamethasone effects (not shown).

### 3.4. TNF and IL-1β Levels in Colonic Tissues

Colonic inflammation induced by DNBS was associated with a significant increase in tissue TNF levels (11.5 ± 0.52 pg/mg tissue) compared to the values obtained in the control animals (3.8 ± 0.9 pg/mg tissue). Treatment with AR170 or DEX significantly decreased the concentration of this cytokine in colonic tissues (Figure 4B).

Rats with colitis displayed a significant increase in colonic IL-1β levels (196.1 ± 36.3 ng/mg tissue) compared to the control animals (45.9 ± 21 ng/mg tissue). Treatment with AR170 and DEX was associated with a significant decrease in IL-1β levels (Figure 4C). The effects of AR170 on tissue TNF and IL-1β were counteracted by MRS1523 (Figure 4C). In contrast, the A_3_AR antagonist did not alter the dexamethasone effect (not shown).

### 3.5. Effect of the Acute and Repeated Treatment with AR170 on the Visceral Pain Induced by DNBS

**Figure 5:**
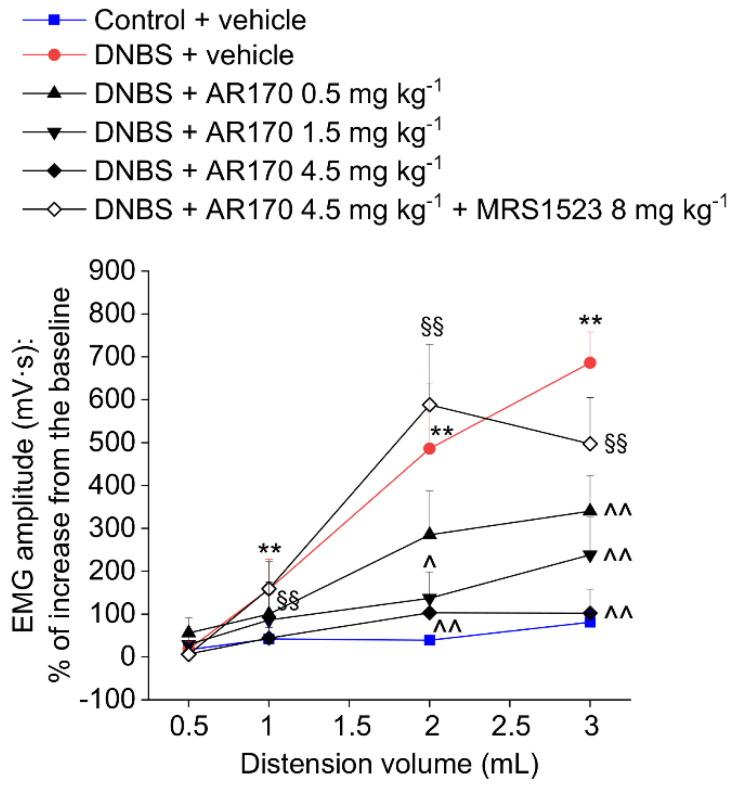
Visceromotor response (VMR) to the colorectal distension of DNBS treated animals after the acute administration of AR170 (0.5–4.5 mg/kg i.p.) both in the absence and the presence of the selective A_3_AR antagonist MRS1523 (8 mg/kg i.p., injected 15 min before AR170). Tests were performed 14 days after DNBS injection and 15 min after the acute administration of AR170. Control animals received the vehicle. Each value represents the mean ± S.E.M. (*n* = 4). * *p* < 0.05 and ** *p* < 0.01 vs. the control + vehicle treated animals. ^ *p* < 0.05 and ^^ *p* < 0.01 vs. DNBS + vehicle treated animals. ^§§^ *p* < 0.01 vs. DNBS + AR170 4.5 mg kg^−1^ treated animals.

**Figure 6:**
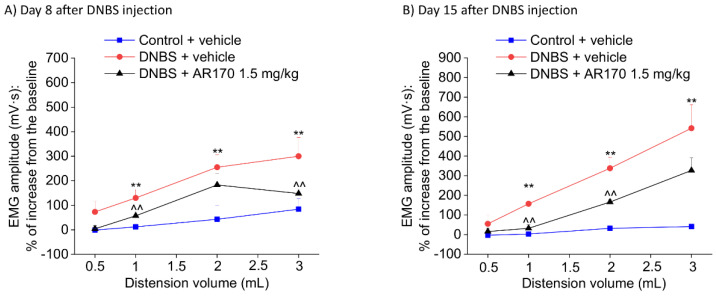
Visceromotor response (VMR) to the colorectal distension of DNBS-treated animals after the repeated administration of AR170 (1.5 mg/kg/day). The test was performed on day 8 (**A**) and 15 (**B**) after DNBS injection, 24 h after the last treatment. Control animals received the vehicle. Each value represents the mean ± S.E.M. (*n* = 4). * *p* < 0.05 and ** *p* < 0.01 vs. the control + vehicle treated animals. ^ *p* < 0.05 and ^^ *p* < 0.01 vs. DNBS + vehicle treated animals.

The measurement of the VMR to colorectal distension was used to assess the visceral sensitivity alterations in rats. Colorectal distension was carried out by inflating the balloon positioned in the colon with increasing volumes (0.5–3 mL). After DNBS injection, the VMRs induced by 1, 2, and 3 mL were significantly higher than those of the controls (Figure 5 and Figure 6). This visceral hypersensitivity was established in concomitance with intestinal inflammation (day 8, Figure 6A) and persisted in the remission phase of colitis (day 14, Figure 5 and Figure 6B), as previously reported in the literature [21]. On day 14, the acute administration of AR170 (0.5, 1.5, and 4.5 mg/kg) dose-dependently relieved the visceral hypersensitivity induced by DNBS. The highest dose (4.5 mg/kg) completely reversed the sensitive alterations back to the values of the controls. AR170 1.5 mg/kg significantly reduced the VMR of the animals to CRD (2–3 mL), while the lowest dose (0.5 mg/kg) was only partially effective, significantly lowering the VMR only in response to a 3 mL stimulus. Pre-treatment with the selective A_3_AR antagonist MRS1523 (8 mg/kg) [26] completely abolished the acute pain-relieving effects of AR170 (4.5 mg/kg).

Relying on the acute pain-relieving efficacy shown by AR170 1.5 mg/kg in the previous tests, we chose this dose for the following experiment, in which the therapeutic effect of the repeated administration of AR170 on the visceral pain induced by DNBS was examined. Repeated treatment with AR170 (1.5 mg/kg) was able to counteract the development of visceral hypersensitivity induced by colitis in rats. On day 8, during the acute inflammatory phase, DNBS animals treated with the A_3_AR agonist showed a significant decrease of their abdominal response to both 1 and 3 mL (Figure 6A). This effect was maintained in the remission phase of colitis (day 15, Figure 6B) when the VMR to both 1 and 2 mL significantly decreased in the DNBS animals receiving AR170. Even the response to 3 mL appeared to be reduced on day 15, despite not reaching statistical significance (Figure 6B).

## 4. Discussion

The pivotal role played by adenosine in regulating the inflammatory responses and counteracting tissue injury through the engagement of specific receptors is widely recognized [7,27,28]. The pharmacological modulation of the adenosine receptor subtype A_2A_ and A_3_ ARs have demonstrated their beneficial effects in several models of experimental colitis, thereby driving the scientific community to develop novel and selective ligands for these receptor subtypes as promising tools to manage IBDs [16,29,30,31,32,33]. Unfortunately, the great expectations for applying A_2A_AR agonists in clinical practice are challenged by the severe side effects of such ligands at the cardiovascular level [34]. Hence, increasing attention is being placed on the pharmacology of A_3_AR.

This receptor subtype is upregulated in activated immune cells, including neutrophils, monocytes, macrophages, dendritic cells, lymphocytes, splenocytes, bone marrow cells, and mast cells [10]. Once pharmacologically activated, A_3_AR exerts a marked immunosuppressive effect associated with a safe and well tolerated profile, as reported in preclinical studies and in Phase I and II human clinical studies [10].

For these reasons, today, A_3_AR is considered a novel and very promising therapeutic target, from which new agonists characterized by improved dynamic and kinetic properties are being designed. In this regard, AR170 is of great interest due to its high A_3_AR affinity (K_i_ A_3_AR = 0.44 nM in the radioligand binding assay) and remarkable selectivity versus other AR subtypes (i.e., about 97,000-fold selectivity versus A_2A_AR) [17,18], making it among the most potent and selective A_3_AR agonists reported to date.

Presently, the pharmacological management of IBD patients is far from satisfactory. Usually, the targeted treatments for the inflammatory aspect of these diseases are unsatisfactory, and the modulation of visceral hypersensitivity is ineffective.

Visceral pain is a critical component of IBD, which often persists even after complete resolution of the inflammation, significantly impacting the well-being of the patients [35,36,37]. Based on these findings, we developed the present study to investigate the putative anti-inflammatory and pain-relieving effects of AR170 in a rat model of colitis. To pursue the above aims, the effects of AR170 were assayed in a rat model of colitis elicited by DNBS. DEX was used as a glucocorticoid drug with known anti-inflammatory activity to assess the relative anti-inflammatory potency of the A_3_AR ligand.

DNBS-induced colitis is a murine model reminiscent of human Crohn’s disease, which is characterized by body weight loss, diarrhea, ulceration and bleeding, the depletion of goblet cells, and the formation of granulomas within the gut wall [20]. In parallel, it has been reported that DNBS-induced colitis represents a useful model for studying visceral hypersensitivity in response to colorectal distension [21]. The suitability of this preclinical model to assay both the anti-inflammatory and pain-relieving properties of novel drugs increases the translational potential of this research.

In the present study, AR170 administration was associated with significant improvements in all tissue inflammatory parameters, including colon length macroscopic scores, tissue cytokines (TNF and IL-1β), and tissue polymorphonuclear neutrophils and macrophage infiltration (MPO). Consistent with the data presented here, A_3_AR agonists were previously evaluated and shown to be effective in experimental models of inflammatory intestinal disorders [12,29,31]. In particular, it was reported in murine DSS colitis that the activation of A_3_AR, expressed in colonic epithelia, exerts anti-inflammatory activity through the inhibition of the pro-inflammatory cytokine TNF and IL-1β via the inhibition of the NF-κB signaling pathways [12]. Recently, these preclinical data have been substantiated by Ren et al. [11]. In this study, the pharmacological stimulation of A_3_AR, via Cl-IB-MECA, decreased TNF and IL-1β production and attenuated NF-κB p65 activation in colonic tissues in patients with ulcerative colitis, thus corroborating the use of A_3_AR agonists as an efficacious treatment for IBD patients [11].

Although abdominal pain is an important patient-reported outcome for the evaluation of therapeutic efficacy [38,39], most treatments for Crohn’s disease are primarily focused on inflammation control and only marginally address the problem of chronic abdominal pain [40]. Moreover, opioid treatment, the current frontline therapy for abdominal pain, has serious complications and produces a condition called narcotic bowel syndrome, which leads to an exacerbation of pain rather than relief [41,42].

Over the years, increasing interest has been focused on characterizing the molecular mechanisms involved in visceral hypersensitivity to identify novel targets for managing abdominal pain [43,44,45,46,47]. The adenosine system was also previously demonstrated to have an important role in pain signaling [48,49]. As a result, A_3_AR modulation emerged as an effective strategy for the treatment of chronic pain with different etiologies, as attested by several preclinical studies [15,50,51,52,53].

In addition to its anti-inflammatory properties, the present work demonstrated the protective effects of AR170 on the development of visceral pain induced by DNBS in animals, as observed during both the acute phase of colitis and remission. The protective efficacy of AR170 on the development of visceral pain after DNBS injection was comparable to that shown by DEX in a previous study conducted by our research group using the same preclinical model of colitis [21]. However, while the pain-relieving effect of DEX is likely attributable to its anti-inflammatory activity and the resulting prevention of intestinal damage, AR170 seems to directly modulate visceral pain signaling. Notably, the mediation of pain by AR170 was detected after the repeated administration of a lower dose (1.5 mg/kg) compared to that used for obtaining protection from intestinal damage (4.5 mg/kg), suggesting the involvement of A_3_AR in the regulation of visceral sensitivity. A_3_AR activation mediated by AR170 may limit excitatory neurotransmission, which is altered in visceral pain [54,55,56]. Numerous results in the literature demonstrate neuroprotective effects related to A_3_AR activation and the resulting decrease in neuronal excitability [15,57,58,59,60]. This mechanism could be adjuvant to the anti-inflammatory properties, actively contributing to the therapeutic effects of AR170 on visceral pain. Notably, previous evidence [15] demonstrating the modulatory role of adenosine A_3_ receptors in the regulation of colonic neuromuscular functions in the presence of bowel inflammation led researchers to hypothesize the potential application of the A_3_AR agonist as a suitable tool for the management of IBD patients characterized by enhanced bowel motor activity and diarrhea.

AR170 has also proven effective in the acute relief of visceral pain, showing additional advantages over the use of steroidal and non-steroidal anti-inflammatory drugs [61]. These data agree with the anti-hyperalgesic effects previously reported for other A_3_AR agonists [14,15], supporting the hypothetical direct modulation of visceral pain transmission, which may represent a further benefit of AR170 in the therapy of bowel diseases.

## 5. Conclusions

Overall, the present results suggest that the pharmacological modulation of A_3_AR represents a novel and appealing therapeutical strategy for the management of inflammatory bowel disorders, as this method is contextually able to dampen the inflammatory process and mitigate the visceral hypersensitivity associated with colitis.

## Figures and Tables

**Table 1:** Criteria for scoring macroscopic colonic ulceration and inflammation.

Score	Appearance
	Macroscopical
0	Normal
1	Localized hyperemia, no ulcers
2	Ulceration without hyperemia or bowel wall thickening
3	Ulceration with inflammation at one site
4	2 or more sites of ulceration and inflammation
5	Major sites of damage extending >1 cm along length of colon
6	When an area of damage extended >2 cm along length of colon. Score was increased by 1 for each millimeter of bowel wall thickness
